# Oxysterols Suppress Release of DNA from Granulocytes into Extracellular Space After Stimulation with Phorbol Myristate Acetate

**DOI:** 10.3390/biomedicines12112535

**Published:** 2024-11-06

**Authors:** Yuichi Watanabe, Takashi Obama, Tomohiko Makiyama, Hiroyuki Itabe

**Affiliations:** Division of Biological Chemistry, Department of Pharmaceutical Sciences, Showa University Graduate School of Pharmacy, 1-5-8 Hatanodai, Shinagawa-ku, Tokyo 142-8555, Japan; yuichi.watanabe.yw@gmail.com (Y.W.); obama@pharm.showa-u.ac.jp (T.O.); t-maki@pharm.showa-u.ac.jp (T.M.)

**Keywords:** neutrophil extracellular trap, oxysterol, HL-60, methyl-β-cyclodextrin

## Abstract

Background: Neutrophils eject their DNA strings and cellular proteins into the extracellular space upon treatment with various stimulants. In the present study, we examined the effects of four major oxidized cholesterol metabolites on DNA release from granulocytes. Methods and Results: When oxysterols were added to HL-60-derived granulocytes stimulated with phorbol 12-myristate 13-acetate (PMA), they suppressed the release of DNA and myeloperoxidase from the cells. Among the four oxysterols tested, 7-ketocholesterol was the most effective. Addition of the same concentration of 7-ketocholesterol did not induce any cytotoxic effects, as evaluated based on the release of lactate dehydrogenase and 3-(4,5-dimethylthiazol-2-yl)-2,5-diphenyl-2H-tetrazoliumbromide (MTT) assays. DNA release from human peripheral blood neutrophils after PMA stimulation was also suppressed by 7-ketocholesterol. Liquid chromatography with tandem mass spectrometry (LC-MS/MS) analysis was used to quantify sterol content in the cells. The addition of oxysterols increased the cellular content of the corresponding compounds by more than 10-fold compared to those at baseline. Treatment of HL-60-derived granulocytes with methyl-β-cyclodextrin that removes sterol compounds from the membranes increased DNA release from the cells in a dose-dependent manner. Conclusions: These results suggest that oxysterols have suppressive effects on DNA release from granulocytes stimulated with PMA.

## 1. Introduction

Neutrophil extracellular trap (NET) formation is a self-defense response in which neutrophils eject DNA strings into the extracellular space [1]. Upon stimulation with phorbol 12-myristate 13-acetate (PMA) or various pathogen-related materials, such as formyl-methionyl-leucyl-phenylalanine, decondensation of chromatin, and rupture of the nuclear membrane, DNA strings together with various cell proteins, such as histones, neutrophil elastase, and myeloperoxidase (MPO), spread out from the cells [2]. When DNA strings and these proteins spread in infected tissues, they serve as effective bactericidal nets. Therefore, NET formation acts as a natural self-defense system. However, DNA and histones are well-known damage-associated molecular patterns (DAMPs), and DNA and proteins released from cells also act as inducers of inflammatory responses involved in various diseases [3], including rheumatoid arthritis [4], thrombosis [5], and atherosclerosis [6].

NETs were initially found as anti-bacterial self-defense system of neutrophils. Currently, NETs are classified into three types depending on the morphological changes in cells: vital NETs, mitochondrial NETs, and suicidal NETs [7]. In vital NET formations, through stimulation with *Staphylococcus aureus*, NETs are released from the cells through nuclear budding and vesicular release. Cells release DNA without disruption of the plasma membrane and cellular death; however, the cells become anuclear [8,9]. Mitochondrial NETs are induced through pretreatment of neutrophils with GM-CSF and subsequent stimulation with LPS or complement factor 5a. Under these conditions, neutrophils release mitochondrial DNA, but not nuclear DNA, as extracellular traps [10]. When neutrophils are stimulated with phorbol 12-myristate 13-acetate (PMA), decondensed DNA is released from the cells through membrane perforation. Such a lytic process of NET formation is called suicidal NETs [9].

DNA release similar to NET formation was also observed in some granulocytes, such as monocytes and eosinophils [11]. Thus, the words ETs and ETosis can be used to refer to DNA release from various cells and the cell death associated with DNA release of these cells.

Cholesterol is oxidized either enzymatically or non-enzymatically under physiological conditions to generate various oxysterols [12,13]. Bile acids are the major metabolites of cholesterol that are generated in the liver and secreted into the bile juice. In the first step of bile acid formation, cholesterol is hydroxylated to form 7α-hydroxycholesterol (7αHC) [14]. In the neural tissues, cholesterol is converted to 24-hydroxycholesterol (24HC), which is released into the bloodstream and transferred to the liver [15]. Cholesterol is also oxidatively modified non-enzymatically through interactions with free radicals, both in vivo and in vitro. It is well-known that the major oxidized product of cholesterol in oxLDL is 7-ketocholesterol (7KC), and it is a cytotoxic product [16]. Apoptosis of endothelial and smooth muscle cells, but not of human fibroblasts, was induced by 7KC [17]. Apoptosis of neuronal cells was induced by 7KC through activation of the nuclear factor–kappa B (NF-kB) and Akt pathways [18]. Some oxysterols, including 24HC and 27-hydoxycholesterol (27HC), are ligands of the liver X receptor (LXR), and 25-hydroxycholesterol (25HC) induces LDL receptors via the activation of the sterol regulatory-element binding protein (SREBP)-SREBP cleavage activating protein (SCAP) system [19]. Oxysterols were shown to accumulate in atherosclerotic plaques [20]. In addition, the plasma level of 7KC was reported to be associated with risk of cardiovascular events and total mortality based on a prospective follow-up study [21].

Several oxidative products have been reported to be involved in enhancing DNA release from cells. For example, oxidized phospholipids, such as oxidized phosphatidylethanolamine [22] and oxidized phosphatidylcholine [23], have been reported to enhance NET formation. Oxidized low-density lipoprotein (oxLDL), which is known as a source of oxidized phospholipids in vivo, was shown to enhance PMA-induced NET formation in neutrophils [24]. In addition to the oxidation products of phospholipids, 7KC and other oxysterols are generated in oxidized LDL [25], and the cytotoxic effects of these oxysterols have been reported in various cells, including vascular smooth muscle cells [17,26]. However, the effect of oxysterols that could affect membrane stability in NETosis has not been fully investigated.

Cholesterol and its metabolites are essential components that affect the membrane stiffness of the cell membrane [27]. We postulated that membrane components may affect NET formation because disruption of the plasma and nuclear membranes is accompanied by DNA release from neutrophils. Thus, we assumed that oxysterols, such as 7KC, might activate NET formation. In the present study, we examined the effects of four oxysterols on the release of DNA and proteins from neutrophils. Unexpectedly, oxysterols, but not cholesterol, suppressed DNA release from neutrophils. Furthermore, treatment of cells with methyl-β-cyclodextrin (MβCD) that extracts sterol compounds from cell membranes to form inclusion complexes enhanced DNA release from PMA-stimulated cells. These observations suggest that oxysterols may act as modulators of neutrophil activation.

## 2. Materials and Methods

### 2.1. Materials

The following biological materials were procured commercially: 7α-hydroxycholesterol (7αHC) was from FOCUS Biomolecules (Plymouth Meeting, PA, USA); 7-ketocholesterol (7KC) and 25-hydroxycholesterol (25HC) were from Cayman Chemicals (Ann Arbor, MN, USA); 27-Hydroxycholesterol (27HC) was from Sigma-Aldrich (St. Louis, MO, USA); deutelium-labeled 7-ketocholesterol-[d_7_] (7KC-d_7_) was from Santa Cruz Biotechnology (Santa Cruz, CA, USA); Micrococcal nucleases were from Takara Bio Inc. (Shiga, Japan); polyclonal rabbit anti-myeloperoxidase (MPO; A0398) antibody was from Dako (Carpinteria, CA, USA); polyclonal rabbit anti-glyceraldehyde-3-phosphate dehydrogenase (GAPDH; G9545) antibody was from Sigma-Aldrich (St. Louis, MO, USA); SYTOX Green was from Thermo Fisher Scientific (Waltham, MA, USA); the Cytotoxicity LDH Assay Kit-WST was from Dojindo (Kumamoto, Japan); and the MTT Cell Count Kit was from Nacalai Tesque (Kyoto, Japan). All other chemicals were of analytical grade and were obtained from Sigma-Aldrich, Fuji Film Wako (Osaka, Japan), or Nacalai Tesque (Kyoto, Japan).

### 2.2. Cell Culture and Cell Treatment

Peripheral neutrophils were isolated from freshly drawn blood of healthy donors, as previously reported [28]. HL-60 cells (obtained from the American-Type Culture Collection) were maintained in Roswell Park Memorial Institute-1640 medium (RPMI-1640) supplemented with 5% (*v*/*v*) fetal bovine serum (FBS), 100 U/mL of penicillin, and 100 μg/mL of streptomycin. All cell cultures were maintained in a humidified incubator at 37 °C under an atmosphere of 5% CO_2_. To differentiate the cells into neutrophil-like cells, HL-60 cells were cultured in the presence of 2 µM of all-*trans*-retinoic acid (AtRA) in RPMI-1640 supplemented with 5% (*v*/*v*) FBS for four days following the procedure described previously [23]. Differentiation of cells was checked based on increases in the gene expression of *ITGAM* and *FCER1A* (genes for CD11b and Fcε receptor, respectively, Supplementary Materials). Because we did not examine specific markers for neutrophil differentiation, we describe the HL-60-derived cells in this study as granulocytes. Differentiated cells were recovered, resuspended in serum-free RPMI-1640, and used in the experiments.

### 2.3. Measurement of Released DNA

The released DNA was quantified as described in our previous study, with slight modification [24]. Differentiated HL-60 cells were seeded in a 24-well plate (5.0 × 10^5^ cells/well) and incubated for 30 min at 37 °C. Cells were treated with the indicated concentrations of either one of the oxysterols for 30 min, and DNA release was induced through an additional 2 h of incubation with 25 nM of PMA. Human peripheral blood neutrophils were seeded in a 24-well plate (5.0 × 10^5^ cells/well) precoated with poly-L-lysine and incubated for 30 min at 37 °C. Cells were treated with 7KC, cholesterol, or vehicle for 30 min, and DNA release was induced through an additional 2 h of incubation with 5 nM of PMA. For the experiments with sterol extraction, up to 100 μM of methyl-β-cyclodextrin (MβCD) was added to the cells 30 min before the beginning of the 2 h of incubation with 25 nM of PMA. Thereafter, the samples were treated with 1 U/mL of Micrococcal nuclease for 10 min at 37 °C to degrade the released DNA. The debris were removed through centrifugation at 1800× *g* for 10 min, and the supernatant was mixed with 1 µM of SYTOX Green^®^. The fluorescent intensity was measured using Varioskan^®^ Flash (Thermo Fisher Scientific).

### 2.4. LC-MS/MS

Differentiated cells were harvested by removing the culture medium with centrifugation at 1500× *g* for 5 min. After the cell pellet was washed with phosphate-buffered saline (PBS), the cells were resuspended in PBS and split into two tubes: one for measuring sterol content through liquid chromatography with tandem mass spectrometry (LC-MS/MS) and the other for measuring cellular proteins. After removing the PBS, the cellular lipids were extracted using hexane/2-propanol (3:2, *v*/*v*). As an internal standard, 600 ng of 7KC-d_7_ was added to the samples. Samples were dried under a nitrogen gas stream, and the lipids were re-dissolved in 95% (*v*/*v*) acetonitrile and centrifuged at 20,000× *g* for 15 min to remove insoluble matter. Oxysterols and cholesterol were quantified through LC-MS/MS analysis using atmospheric pressure chemical ionization (APCI), as cholesterol is not efficiently ionized through electrospray ionization (ESI) [29]. LC-MS/MS was performed using QTRAP6500+ with APCI (SCIEX, Tokyo, Japan) equipped with an InertSustain C18 HP column (3 µm, 2.1 × 100 mm: GL Science, Tokyo, Japan). Five microliters of the sample was injected using an autosampler. Solvent A was ultrapure water containing 0.1% (*v*/*v*) formic acid, and solvent B was acetonitrile containing 0.1% (*v*/*v*) formic acid. The column oven was set at 40 °C. The flow rate was 0.2 mL/min. A multistep gradient elution was performed using the following 25 min gradient profile (min/% B): 0 min/75% B–15 min/100% B–23.9 min/100% B–24 min/75% B–25 min/75% B. The APCI source parameters were as follows: source temperature 300 °C, nebulizer current 2, and curtain gas 45 psi. The optimized conditions of mass spectrometry for the detection of cholesterol and oxysterols are listed in Table 1. Although hydroxycholesterols have the same molecular weight, they can be specifically detected based on the difference in retention time and the combination of precursor and product ions.

For protein content measurement, the PBS-free cells were lysed in lysis buffer (50 mM of Tris–HCl, pH 8.0, 1 mM of EDTA, 150 mM of NaCl, 1% NP-40, and 0.25% sodium deoxycholate). The lysate was centrifuged at 18,000× *g* for 5 min at 4 °C to remove cellular debris. The amount of protein in the lysate was measured using the BCA assay.

The sterol content was calculated based on the internal standard and normalized to the cell protein.

### 2.5. Cytotoxicity Determination Through LDH Assay

Differentiated HL-60 cells were seeded into a 24-well plate (5.0 × 10^5^ cells/well) and incubated at 37 °C for 30 min. After the cells were treated with 5 µM of 7KC or 2 mM of H_2_O_2_ for 2.5 h, the cell suspension was centrifuged at 250× *g* for 2 min, and the supernatant was used for determination of lactate dehydrogenase (LDH) released from cells using a Cytotoxicity LDH Assay Kit-WST (Dojindo) according to the manufacturer’s protocol.

### 2.6. Cell Viability Measurement Through MTT Assay

Differentiated HL-60 cells were seeded in a 96-well plate (2.5 × 10^4^ cells/well) and incubated for 30 min at 37 °C. After the cells were treated with 5 µM of 7KC or 2 mM of H_2_O_2_ for 2.5 h, viable cells were determined using a 3-(4,5-dimethylthiazol-2-yl)-2,5-diphenyl-2H-tetrazoliumbromide (MTT) assay (MTT Cell Count Kit; Nacalai Tesque) according to manufacturer’s protocol.

### 2.7. Statistical Analysis

Data are presented as the mean ± standard deviation (SD). Statistical significance was calculated using a one-way analysis of variance with Tukey’s post hoc test. Statistical significance was set at *p* < 0.05. Easy R (EZR) version 1.61 (The R Foundation for Statistical Computing) was used for statistical analyses [30].

## 3. Results

### 3.1. Oxysterols Suppressed the Release of DNA and MPO from Granulocytes

**Figure 1:**
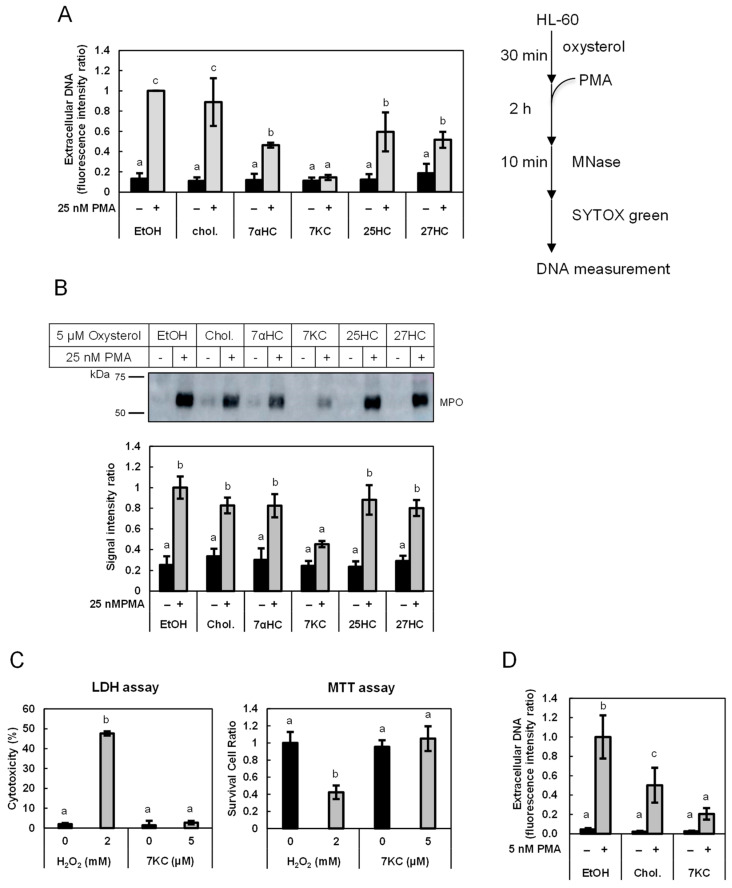
Release of DNA and MPO from granulocytes was suppressed by oxysterols. (**A**,**B**) HL-60-derived granulocytes were treated with 5 µM of cholesterol (chol.) or oxysterols dissolved in ethanol or vehicle (EtOH) for 30 min and subsequently stimulated with 25 nM of PMA for 2 h. (**A**) The amount of DNA released from the stimulated cells into the culture medium was measured using SYTOX Green assay. (**B**) The amount of released MPO was detected through Western blot. The band intensity was calculated using ImageJ software. (**C**) HL-60-derived granulocytes were treated with 5 µM of 7KC or 2 mM of H_2_O_2_ for 2.5 h. The cytotoxic effect of 7KC was evaluated based on LDH release, and the effect of 7KC on cell viability was evaluated based on the MTT assay. As a positive control, cells were treated with 2 mM of H_2_O_2_. The final concentration of ethanol in the medium was 0.1%. (**D**) Human peripheral blood neutrophils were treated with 5 µM of cholesterol (chol.) or 7KC dissolved in ethanol or vehicle for 30 min and subsequently stimulated with 5 nM of PMA for 2 h. The data show mean ± SD obtained in three independent assays. The different lowercase letters indicate significant differences (*p* < 0.05) ((**A**,**B**) relative to EtOH with 25 nM of PMA; (**C**) MTT assay, 0 mM of H_2_O_2_). PMA: phorbol 12-myristate 13-acetate; MPO: myeloperoxidase; Chol: cholesterol; 7αHC: 7α-hydroxycholesterol; 7KC: 7-ketocholesterol; 25/27HC: 25/27-hydroxycholesterol; LDH: lactate dehydrogenase; MTT: 3-(4,5-dimethylthiazol-2-yl)-2,5-diphenyl-2H-tetrazoliumbromide; SD: standard deviation.

Human promyelocytic leukemia HL-60 cells were differentiated into granulocytes through incubation with AtRA for four days. Differentiated HL-60 cells were stimulated with 25 nM of PMA to induce the release of DNA and MPO from the cells. Incubation of differentiated HL-60 cells with 5 µM of either cholesterol or four types of oxysterols did not affect the release of DNA. The addition of either one of four oxysterols, but not cholesterol, significantly reduced the level of PMA-induced DNA released from the cells into the culture medium (Figure 1A). Among the four oxysterols, 7KC almost completely inhibited DNA release. In the absence of PMA stimulation, incubation of the cells with 5 µM of either cholesterol or the oxysterols did not affect the release of DNA. It is well-known that cells release various cellular proteins, such as MPO, in addition to DNA during NET formation. Stimulation of cells with 25 nM of PMA increased the extracellular MPO, and 5 µM of 7KC significantly decreased the MPO release. Other oxysterols were less effective than 7KC at inhibiting DNA release and did not reach statistical significance in the reduction of extracellular MPO under these conditions (Figure 1B).

It is widely recognized that 7KC has cytotoxic effects. To check for cell damage caused by 7KC under the current experimental conditions, the activity of lactate dehydrogenase (LDH) released into the medium was measured. Incubation of HL-60-derived granulocytes with 5 µM of 7KC for 2.5 h showed little LDH release from cells, whereas 2 mM of H_2_O_2_ for 2.5 h, used as a positive control, resulted in a marked increase in LDH release, indicating that this concentration of 7KC does not destroy the cell membranes. The viability of differentiated HL-60 cells after 2.5 h incubation with 5 µM of 7KC was quantified through the MTT assay. Similarly to the results of the LDH assay, incubation with 2 mM of H_2_O_2_ for 2.5 h resulted in a 60% decrease in cell viability, while 5 µM of 7KC did not reduce the cell viability (Figure 1C). Taken together, these results suggested that 7KC is not cytotoxic to differentiated HL-60 cells at this concentration.

Differentiated HL-60 cells are widely used as neutrophil-like cells; however, the responses to various stimulants are slightly different from circulating neutrophils. We examined the effect of 7KC on DNA release from human neutrophils recovered from peripheral blood. As shown in Figure 1D, 7KC suppressed the DNA release by 80% in PMA-stimulated neutrophils. Cholesterol partially suppressed DNA release from human peripheral neutrophils after PMA stimulation. The effect of cholesterol on peripheral neutrophils may suggest some difference in the susceptibility between cell membranes of peripheral neutrophils and differentiated HL-60 cells.

### 3.2. Pre-Treatment of Oxysterols Caused Significant Increase in Cellular Oxysterols in Differentiated HL-60 Cells

**Figure 2:**
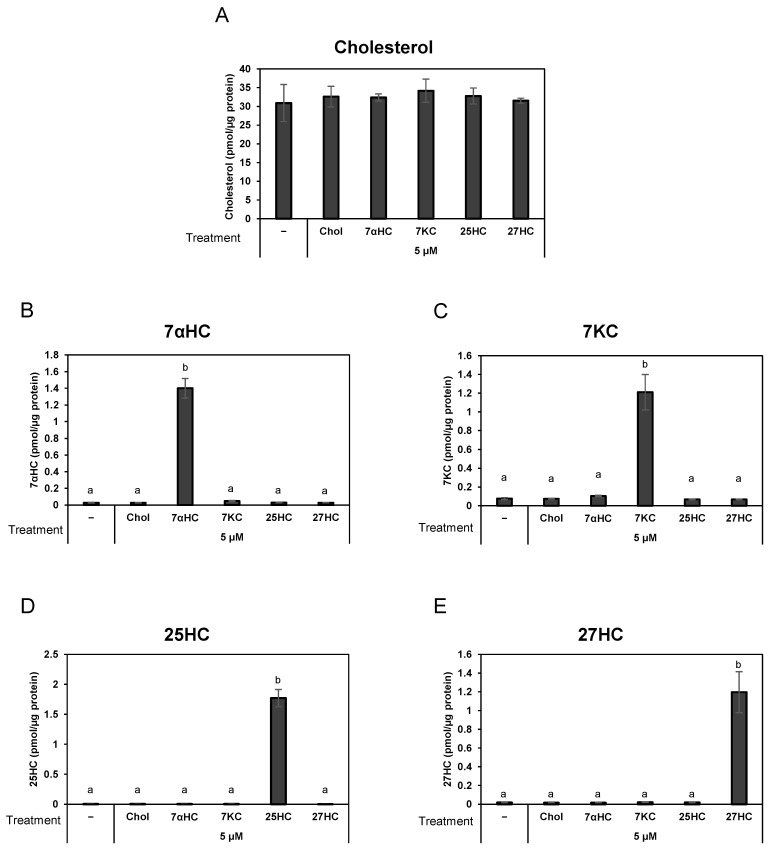
The levels of cellular sterols in cells treated with indicated sterols. HL-60-derived granulocytes were treated with or without 5 µM of either one of the sterol compounds (cholesterol, 7αHC, 7KC, 25HC, or 27HC) for 2 h at 37 °C. After the medium was discarded, cells were extracted with hexane:2-propanol (3:2 *v*/*v*). The amounts of the five sterols in each sample were measured using LC-MS/MS with the analytical conditions listed in Table 1. The panels show the amounts of sterols (cholesterol (**A**), 7αHC (**B**), 7KC (**C**), 25HC (**D**), and 27HC (**E**)) in the cells pretreated with either one of these sterols. The data shows mean ± SD of the amount of sterol per microgram of cellular protein obtained from three independent assays. The different lowercase letters indicate significant differences (*p* < 0.05). Chol: cholesterol; 7αHC: 7α-hydroxycholesterol; 7KC: 7-ketocholesterol; 25/27HC: 25/27-hydroxycholesterol; LC-MS/MS: liquid chromatography with tandem mass spectrometry.

To verify whether the cellular levels of sterols changed after the addition of oxysterols under the experimental conditions, we introduced an LC-MS/MS procedure that selectively quantifies oxysterols and cholesterol. The amounts of sterols in the cells were measured and normalized to the amount of cell protein. Differentiated HL-60 cells contained approximately 30 pmol/µg protein of cholesterol and less than 0.1 pmol/µg protein of oxysterols in the basal condition. Cells were incubated with 5 µM of either one of the four oxysterols or cholesterol for 2.5 h, which is the same duration as that of DNA release. Treatment with one of these oxysterols significantly increased the cellular levels of the corresponding oxysterol, while the other oxysterols or cholesterol remained unchanged (Figure 2). These results suggested that exogenous oxysterols are sufficient to alter the amount of oxysterol in the cells. However, cellular cholesterol levels did not change significantly after treatment with cholesterol (Figure 2A). Presumably, the amount of cholesterol added from exogenous sources is a minor fraction of the total cholesterol in cells.

### 3.3. Pre-Treatment with Methyl-β-Cyclodextrin Facilitated DNA Release

**Figure 3:**
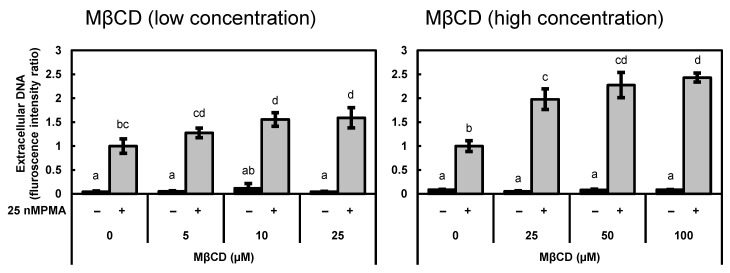
Treatment with methyl-β-cyclodextrin facilitated DNA release from PMA-stimulated cells in a dose-dependent manner. HL-60-derived granulocytes were treated with the indicated concentrations of MβCD for 30 min and subsequently stimulated with 25 nM of PMA for 2 h. The level of DNA release from the stimulated cells into the culture medium was measured using a SYTOX Green assay. The data show mean ± SD obtained in three independent assays. The different lowercase letters indicate significant differences (*p* < 0.05) (relative to 0 µM of MβCD with 25 nM of PMA). MβCD: methyl-β-cyclodextrin; SD: standard deviation; PMA: phorbol 12-myristate 13-acetate.

The addition of oxysterols to HL-60-derived granulocytes caused an increase in cellular sterols and concomitantly suppressed DNA and MPO release. Based on these results, we hypothesized that decreased cellular sterol levels affect cellular responses. To verify this possibility, we examined the effect of the removal of sterols from HL-60-derived neutrophils on DNA release using MβCD before PMA stimulation. As shown in Figure 3, the treatment with MβCD accelerated PMA-induced DNA release in a dose-dependent manner. The DNA release level was quite low even at the highest concentration of MβCD treatment without PMA stimulation, indicating that MβCD alone did not induce DNA release.

## 4. Discussion

In the present study, we found that low concentrations of 7KC and other oxysterols, but not cholesterol, suppressed the release of DNA and MPO in PMA-stimulated HL-60-derived granulocytes. Furthermore, DNA release was accelerated by the removal of (oxy)sterols from the cell membranes following treatment with MβCD. DNA release from human neutrophils was also suppressed by 7KC. These observations suggest that oxysterols suppress PMA-induced DNA release from granulocytes.

Numerous studies have elucidated the significance of NETs, or ETs, in various pathological conditions in vivo, including rheumatoid arthritis, thrombosis, atherosclerosis, and other diseases [3,4,5,6,31,32]. Release of DNA and proteins from granulocytes is currently considered a common phenomenon that induces inflammation; therefore, various compounds that affect granulocytes are potentially useful for understanding and regulating inflammatory responses.

We have previously shown that a small amount of oxLDL enhances NET formation in differentiated HL-60 cells and human peripheral neutrophils after stimulation with PMA [23]. These observations suggest that oxidized lipids, which are formed in oxLDL as well as in various diseased tissues, may influence NET formation. Oxidized phospholipids containing truncated 2-acyl chains at the *sn*-2 position of phosphatidylcholine (truncated oxPC) have been studied as the inflammatory oxidation products of oxLDL [33]. We showed that 10 μM of truncated oxPC enhances the release of DNA and MPO from differentiated HL60 cells [24]. Oxysterols, including 7KC, are generated in vivo and thought to be cytotoxic products [13,25,26]. Therefore, it is assumed that the oxysterols may enhance responses like NET formation; however, our observation that oxysterols suppress the release of DNA and MPO in PMA-stimulated neutrophils was unexpected.

Although 7KC was the most effective oxysterol among those tested, it did not show any cytotoxic effects under the experimental conditions. The concentration of oxysterol added to the neutrophils was 5 μM. We assume that this concentration is critical because a previous study [17] reported that 7KC at 40 μg/mL induces apoptosis and necrosis of vascular cells. This concentration of 7KC (40 μg/mL of 7KC is approximately 100 μM) was 20 times higher than that under our experimental conditions. Tabas et al. reported that 50 μg/mL (125 μM) of 7KC induced NET formation in neutrophils from murine blood [34]; however, the concentration of 7KC used here was even higher than that used in the previous study. Therefore, 7KC may have a biphasic property depending on the concentration range; 7KC induced DNA release at high concentrations but suppressed it at low concentrations. Furthermore, the effects of a low concentration of oxysterols on the membrane stiffness of endothelial cells were reported previously. Shentu et al. showed that the elastic modulus of bovine aortic endothelial cells increased when exposed to 10 μg/mL of 7KC or 27HC, indicating an increase in cell stiffness [35]. The effects on membrane stiffness induced by exposure to 7KC or 27HC were reversed through enrichment of the cells with cholesterol [35]. Oxidized LDL increases the stiffness of cell membranes by supplying oxysterols to and removing cholesterol from cell membranes [36]. Our results demonstrated that the addition of 7KC suppressed DNA release from granulocytes. Taken together, 7KC under certain concentration might stabilize the cell membranes, resulting in a decrease in NET formation. In this study, we did not examine the effect of oxysterols on vital NETs under a specific condition; however, it is speculated that stabilization of cell membranes by oxysterols could also suppress the exocytotic release of DNA.

In addition, removal of sterols enhanced DNA release in PMA-stimulated cells. MβCD retrieves sterols from membranes and forms inclusion complexes with retrieved sterols [37,38]. Oh et al. reported previously that treatment of neutrophils with MβCD reduced the deformability of the membranes [39]. Such an effect of sterols may explain the alteration of the susceptibility of cells to PMA-induced membrane damage in our experimental conditions. The ligand of MβCD is not necessarily specific for oxysterols; however, because the basal concentration of oxysterols in differentiated HL-60 cells is almost 1000-fold lower than that of cholesterol, the effect of removing oxysterols may be more profound than that of removing cholesterol. These observations suggest that oxysterols act as endogenous suppressors of NET formation.

Previous studies, including ours, showed that some lipophilic compounds, such as resveratrol, suppress DNA release from differentiated HL-60 cells [40]. Resveratrol was considered to suppress DNA release not through protein kinase C inhibition but rather membrane stabilization because DNA release from cells stimulated with calcium ionophore was also suppressed by resveratrol. Meanwhile, some lipid compounds, such as oxidized phospholipids, increased NET formation [22,24]. There might be some characteristics of the molecular structure that are effective in stabilizing membranes, or these suppressive lipid compounds may interact with certain proteins that relate to the NET formation processes.

A comprehensive review summarizes many studies on oxysterols reporting deleterious effects or beneficial effects on inflammation, oxidative stress, cell death, and so on [41]. For example, 50 μM of 25HC induced IL-8 secretion in U937 human promyelocytic leukemia of neuronal cells; however, 0.1 μM of 25HC decreased LPS-induced IL-1β mRNA and protein and inflammasome activation in cholesterol-25-hydroxylase-defficient bone-marrow-derived monocytes. Generally, beneficial effects are observed with lower concentrations of oxysterols rather than deleterious effects. The responses are diverse, and some of them seem to be indirect responses through signal transduction. Our present observation indicates another unique effect of oxysterols on cellular responses. One possible consideration would be that oxysterols act as physiological regulators at low concentrations under normal conditions but show toxic properties when their concentrations increase to a certain hazardous range. Certainly, more studies are needed to elucidate the behavior and functions of oxysterols in vivo.

A limitation of the present study is that the mechanisms of oxysterol suppression were not elucidated. Oxysterols, including 7KC, are thought to destabilize membranes [42]. However, we do not think that this is unlikely, because the concentrations used to treat neutrophils were much lower than those used in previous experimental conditions [34]. Another point is that not all of the cellular responses of ETosis were examined in this study. However, we detected citrullinated histones and shape changes under electron microscopy of differentiated HL-60 cells after PMA treatment in our previous study [24]. Another point would be that the present study only included a cell culture study. For the next step, an in vitro or clinical study would clarify the effects of oxysterols on physiological and pathological responses. To conclude this issue, the effects of oxysterols on the physicochemical properties of the cellular membranes should be examined. Our observations suggest that oxysterol-sensitive machinery suppresses NET formation by neutrophils. Further work is needed to identify these target molecule(s), which is the next goal of our subsequent study.

## 5. Conclusions

Our experimental data showed that several oxysterol, including 7KC, have a role in suppressing the release of DNA and MPO from PMA-stimulated neutrophils. Although oxysterols are known for cytotoxicity to various cells at high concentrations, current observations suggest that they are biphasic stimulants that act as suppressors of NET formation under low concentrations.

## Figures and Tables

**Table 1:** The optimized conditions of mass spectrometry for the detection of cholesterol and oxysterols.

Sterol	Precursor Ion (*m*/*z*)	Product Ion (*m*/*z*)	Collision Energy (V)	Retention Time (min)
Cholesterol (Chol)	369.348	90.9	103	19.08
7α-hydroxycholesterol (7αHC)	367.284	145.2	29	7.22
7-ketocholesterol (7KC)	401.278	383.3	31	8.16
25-hydroxycholesterol (25HC)	367.291	147.1	33	3.74
27-hydroxycholesterol (27HC)	385.269	161.2	29	4.10
7-ketocholesterol-d_7_ (7KC-d_7_)	408.261	91.1	19	7.98

## Data Availability

The data presented in this study are available at figshare (https://figshare.com).

## Supplementary Materials

The following supporting information can be downloaded at https://www.mdpi.com/article/10.3390/biomedicines12112535/s1, Table S1. Sequences of primers for qPCR. Figure S1. Quantification of mRNA expression levels in HL-60 cells during differentiation. The mRNA expression levels of *ITGAM* and *FCER1A* were measured using appropriate primers through RT-qPCR. The data show the mean ± SD obtained in the biological triplicate assay. The different lowercase letters indicate significant differences (*p* < 0.05).
