# Supplementary material for: Oxysterols Suppress Release of DNA from Granulocytes into Extracellular Space After Stimulation with Phorbol Myristate Acetate
Source: Biomedicines. 2024 Nov 6;12(11):2535. doi: 10.3390/biomedicines12112535 (PMC11592087; doi:10.3390/biomedicines12112535)
Supplement: Supplementary file 1 [file biomedicines-12-02535-s001.zip › biomedicines-3264369-supplementary.pdf]

Supplementary material

Experimental procedure

Cell treatment: To differentiate the cells into neutrophil-like cells, HL-60 cells were cultured in the presence of 2 µM all-*trans*-retinoic acid (AtRA) in RPMI-1640 supplemented with 5% (v/v) FBS for four days. Cells before starting differentiation and cells incubated with AtRA for two or four days were harvested for RT-qPCR.

RT-qPCR: Total RNA from HL-60 cells were extracted using RNA II (Takara, Shiga, Japan) and reverse-transcribed using MuLV reverse transcriptase (Applied Biosystems) according to the manufacturer's protocol. Real-time quantitative PCR was performed with Applied Biosystems StepOnePlus™ real-time PCR system (Thermo Fisher Scientific) using PowerUp SYBR Green Master Mix (Applied biosystems) according to the manufacturer's protocol. All relative mRNA expression levels were normalized with 18S. Primer sequences are listed in Table S1.

Table S1 Sequences of primers for qPCR

Targets	Forward primers (5' to 3')	Reverse primers (5' to 3')
18S	ACCGCAGCTAGGAATAATGGA	GCCTCAGTTCCGAAAACCA
ITAGAM (encoding CD11b)	GCCTTGACCTTATGTCATGGG	CCTGTGCTGTAGTCGCACT
FCER1A	CTCCATTACAAATGCCACAGTTG	TCACGCGGAGCTTTTATTACAG

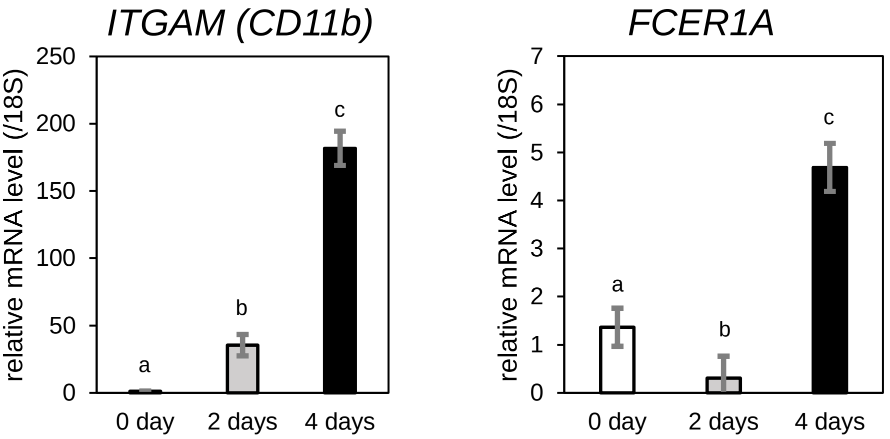

Figure S1. Quantification of mRNA expression levels in HL-60 cells during differentiation. The mRNA expression level of *ITGAM* and *FCER1A* were measured using appropriate primers by RT-qPCR. The data shows mean ± SD obtained in the biological triplicate assay. The different lower-case letters indicate significant differences (P<0.05).
